# Supplementary material for: Dietary Protein Intake in Older Adults from Ethnic Minorities in the Netherlands, a Mixed Methods Approach
Source: Nutrients. 2021 Jan 9;13(1):184. doi: 10.3390/nu13010184 (PMC7827587; doi:10.3390/nu13010184)
Supplement: Supplementary file 1 [file nutrients-13-00184-s001.pdf]

Table S1. Most important protein food sources per ethnic group, based on the contribution to the total daily protein intake

	DUTCH (N = 562)	SOUTH ASIAN SURINAME (N = 319)	AFRICAN SURINAMESE (N = 373)	TURKISH (N = 61)	MOROCCAN (N = 100)
1	Whole wheat bread	Semi skimmed milk	Chicken meat	Lamb, mutton	Moroccan bread
2	Dutch cheese 40+	Whole wheat bread	White rice	Turkish bread	Whole wheat bread
3	Semi skimmed milk	White rice	Dutch cheese 40+	Brown bread	Lamb, mutton
4	Brown bread	Salted and dried cod	Whole wheat bread	Turkish wheat bread	Chicken fillet
5	Dutch cheese 20+	Chicken	Smoked chicken slices	Whole wheat bread	Crustaceans
6	Chicken fillet	Brown bread	Salted and dried cod	Minced beef	Semi skimmed milk
7	Lean beef	Sardines, mackerel, salmon, eel, herring	Brown bread	Veal	Moroccan minced beef
8	Boiled chicken egg	Peanut butter	Nuts, peanuts	Boiled chicken egg	Brown bread
9	Minced beef	Dutch cheese 40+	Dutch cheese 20+	Turkish legumes soup	Boiled chicken egg
10	White pasta	Surinamese legumes	Peanut butter	Legumes	Legumes
